# Efficacy of different exercise modalities for sleep quality in Parkinson’s disease: a systematic review and network meta-analysis

**DOI:** 10.3389/fphys.2026.1854427

**Published:** 2026-06-11

**Authors:** Yejun Zhan, Hongfu Ci, Wei Xue, Zhenyi Ding, Lei Chen

**Affiliations:** 1 College of Education, Lishui University, Lishui, Zhejiang, China; 2 SiChuan Medical Informatics Association, Chengdu, Sichuan, China; 3 Acupuncture and Tuina School, Chengdu University of Traditional Chinese Medicine, Chengdu, Sichuan, China; 4 Department of Neurosurgery, The First People’s Hospital of Shizuishan, Ningxia Hui Autonomous Region, Shizuishan, China

**Keywords:** aerobic exercise, exercise modalities, network meta-analysis, Parkinson’s disease, sleep quality

## Abstract

**Background:**

Sleep disturbances are a common and burdensome non-motor symptom in Parkinson’s disease (PD). The comparative efficacy of different exercise modalities for sleep quality in PD remains unclear. This network meta-analysis (NMA) aimed to compare and rank the effects of various exercise interventions on sleep quality in people with PD.

**Methods:**

This study followed the PRISMA extension statement for network meta-analyses. A systematic search was performed across Web of Science, Embase, PubMed, the Cochrane Library, Scopus, CNKI, and Wanfang databases from their inception to November 3, 2025. Eligible randomized controlled trials (RCTs) were identified, with study selection, data extraction, and bias assessment conducted independently by two reviewers. NMA was performed using Stata 19.0. Consistency was examined using design-by-treatment interaction and node-splitting approaches. Rankings were estimated using the surface under the cumulative ranking curve (SUCRA).

**Results:**

Of 2309 records screened, 16 RCTs involving 932 people with PD were included. Aerobic exercise (AE) significantly improved sleep quality compared with control (SMD = −0.94, 95% CI: −1.82 to −0.07). SUCRA rankings were: AE (highest) > multimodal exercise (MME) > resistance training (RT) > stretching training (ST) > mind-body exercise (MBE) > control. No significant publication bias was found (Egger’s test, P = 0.438).

**Conclusion:**

This NMA indicates that aerobic exercise is the most promising modality for enhancing sleep quality in people with PD and may guide non-pharmacological treatment selection in clinical practice.

**Systematic review registration:**

https://www.crd.york.ac.uk/prospero/, identifier CRD420261341953

## 1 Introduction

Parkinson’s disease (PD) is the second most common neurodegenerative disorder associated with aging, following Alzheimer’s disease (Asadpoordezaki et al., 2025). According to the Global Burden of Disease (GBD) 2021 study, approximately 1.34 million new PD cases were recorded globally in 2021, with projections indicating a rise to 1.93 million incident cases by 2030. Largely driven by population aging, the total number of individuals living with PD is forecasted to increase sharply to 25.2 million by 2050 (Su et al., 2025; Xu L. et al., 2025). The economic impact of this escalating prevalence is considerable; for instance, a study across five European countries found that patients with late-stage PD incur average social costs ranging from 12,156 to 25,649 euros every three months (Kruse et al., 2024). Among the extensive range of symptoms that add to this substantial disease burden, sleep disturbances stand out as one of the most common non-motor manifestations, occurring in more than 80% of individuals with PD. These disturbances tend to worsen progressively with the advancement of the neurodegenerative process (Iranzo et al., 2024). Despite their high prevalence, however, fewer than half of patients report these issues to physicians or receive adequate attention and management (Taximaimaiti et al., 2021). Clinically, sleep issues are typically associated with poorer outcomes, including cognitive decline, mood disorders, and a significantly reduced quality of life for patients (Anderson et al., 2025).

Treatment options for sleep disturbances in PD include both pharmacological and non-pharmacological approaches. Although several studies have confirmed that certain medications (e.g., sedative-hypnotics) offer some efficacy for sleep problems in people with PD, chronic use of these medications is frequently linked to several adverse effects, including dependence, development of tolerance, heightened risk of falls, cognitive impairment, and excessive daytime somnolence (Tang et al., 2024). In contrast, non-pharmacological interventions are increasingly favored due to their lower incidence of adverse effects, relatively sustained benefits, and cost-effectiveness. Among these, exercise-based interventions—such as multimoday exercise (MME), resistance training (RT), mind-body exercise (MBE), stretching training (ST) and others—have demonstrated potential to improve sleep quality in people with PD across multiple randomized controlled trials (Nascimento et al., 2014; Xiao and Zhuang, 2016; Silva-Batista et al., 2017; Abo-Elyazed, 2018; Cheung et al., 2018; Zhu et al., 2019; Amara et al., 2020; Li et al., 2020; Moon et al., 2020; Wu et al., 2021; Wang et al., 2022; Mei-Hua et al., 2023; Li et al., 2024; Mehta et al., 2024; Hu et al., 2025; Li et al., 2025).

Previous meta-analyses have confirmed the overall benefits of exercise on sleep quality in people with PD, yet they either did not differentiate among specific exercise modalities or focused on a broad range of non-pharmacological interventions (including massage and music) without systematically comparing various exercise types (Tang et al., 2024; Li and Hu, 2025). Consequently, the relative efficacy of different exercise modalities—such as aerobic, resistance, stretching, multimodal, and mind−body exercise—remains unknown. However, most existing studies focus on pairwise comparisons between a single exercise modality and a control group, making it challenging to differentiate relative efficacy among the various effective exercise types. This evidence gap hinders clinicians and patients in identifying the optimal exercise regimen for improving sleep outcomes. Therefore, the present study employs a network meta-analysis (NMA) framework to comprehensively assess and compare the effects of various exercise modalities on sleep quality in people with PD. This analysis combines evidence derived from randomized trials to evaluate and rank the comparative effectiveness of various interventions. The goal is to highlight promising exercise strategies and support clinical decision-making for improving sleep quality in PD, which may have implications for patient well-being.

## 2 Methods

This study conducted a systematic review and meta-analysis in accordance with the Preferred Reporting Items for Systematic Reviews and Meta-Analysis (PRISMA) Statement (Hutton et al., 2015). In addition, the review protocol was registered in the PROSPERO registry (CRD420261341953) prior to data extraction.

### 2.1 Search strategy

The following electronic databases—Web of Science, Embase, PubMed, the Cochrane Library, Scopus, China National Knowledge Infrastructure (CNKI), and Wanfang—were systematically searched from their inception until 3 November 2025 to identify all relevant published articles. Search strategies consisted of a combination of Medical Subject Headings (MeSH) terms and free-text keywords related to PD, exercise interventions, and sleep disorders. These included the following: (1) Parkinson’s disease, Parkinson’s, Parkinson disease, exercise, Multimodal exercise, aerobic exercise, balance training, Baduanjin, qigong, Tai Chi, dance, sleep disorders, sleep, sleep quality, etc. The complete search strategies are fully detailed in **Supplementary File 1**.

### 2.2 Eligibility criteria

Eligibility for this review was defined based on the PICOS framework. The following criteria were required for study eligibility: (1) Population: Confirmed diagnosis of Parkinson’s disease. No restrictions were imposed regarding disease stage, age, sex, or time since diagnosis. (2) Intervention: The experimental group participated in an exercise-based intervention of any type. There were no limitations on frequency, duration, intensity, category of exercise, format, setting, or mode of delivery. (3) Comparison: The control group received either a sham activity, a delayed intervention, standard medical care, educational or supportive sessions (e.g., sleep hygiene advice), or a form of physical activity substantially different from the intervention under investigation. Studies that only compared minor variations of similar exercise approaches were excluded. (4) Outcomes: To be included, studies were required to report pre−to−post changes in sleep−related outcomes using at least one of the following instruments: Pittsburgh Sleep Quality Index (PSQI), Parkinson’s Disease Sleep Scale (PDSS), Parkinson’s Disease Sleep Scale−2 (PDSS−2), Epworth Sleepiness Scale (ESS), Mini−Sleep Questionnaire (MSQ), or Insomnia Severity Index (ISI). (5) Study Design: Randomized controlled trials (RCTs) constituted the included studies, irrespective of country of origin or publication type.

Exclusion criteria were as follows: (1) non−randomized study design; (2) Full text irretrievable or data not extractable; (3) The report was only a trial protocol or registration without enrolled participants; (4)The document type was a conference abstract, thesis, dissertation, or literature review; (5) The study duplicated previously published work or shared the same participant cohort as another report—in such cases, the most recent or most comprehensive version was retained; (6) The intervention combined exercise with any non-exercise component (e.g., cognitive training), unless the exercise effect could be isolated.

### 2.3 Data extraction

Two researchers (Z. D. and W. X.) independently extracted the relevant data, covering first author, year, country, sample size, gender, average age, Hoehn and Yahr stage, and disease duration. To assess inter-rater reliability, Cohen’s kappa (κ) was calculated for study selection. They also recorded intervention parameters, including session duration, training frequency, total duration, adherence, supervision, and site. Discrepancies were addressed by discussion, with consultation from a third researcher (Y. Z.) as needed. For each study, mean scores and standard deviations (SDs) were collected for outcome measures to enable effect size calculation. For studies reporting standard errors (SEs) for the experimental and control groups, we calculated standard deviations as SD = SE × $\sqrt{n}$. When SDs and SEs were both missing, we estimated standard deviations from other reported statistics (confidence intervals, t-values, quartiles, ranges, or p-values) using the procedures described in Section 7.7.3 of the Cochrane Handbook. If essential data were still missing after these methods, we contacted the corresponding authors up to four times across six weeks to request the information. The included studies’ characteristics are summarized in **Table 1**.

**Table 1:** Characteristics of the included studies.

Mean age (intervention/control)	Hoehn and Yahr stage	Duration of disease	Intervention detail				Session duration	Training frequency	Duration	Adherence	Supervise	Site	Outcomes
			Intervention group	TEAM	Control group	TEAM							
65.33 ± 8.17 vs 65.82 ± 5.19	2–3	6.0 (3.0–9.0) years vs 3.0 (1.0–7.5) years	RT (leg press, knee extension, chest press, overhead press, pull down) + body weight functional mobility(step-up, squat, jump squat, lunge, side lunge, push-up, assisted pull-up, assisted dip)	RT	Sleep hygiene suggestion	CON	NA	3 times/week	16 weeks	92.2% ± 12.5%, 85% of patients > 90% vs 96.55%	Supervision	Center for Exercise Medicine-base	PSQI, PSGs
67.8 ± 6.8 vs 66.3 ± 8.1	1.7 vs 1.5	5.1 ± 3.9 years vs 4.6 ± 3.7 years	Muscular resistance (bars, medicine balls, thera-bands, bobath balls, ankle weights and barbells were progressively included) + Balance and motor coordination + Aerobic fitness (walk with obstacles)	MME	Routine care	CON	60 min	3 times/week	24 weeks	NA	Supervision	Laboratory-based	Mini-Sleep Questionnaire (MSQ)
63.5 ± 8.5 vs 65.8 ± 6.6	1–3	4.8 ± 2.9 years	yoga	MBE	Wait-list control	CON	60min	2 times/week	12 weeks	NA	Supervision	yoga studio	PDSS
68.83 ± 4.35 vs 67.95 ± 4.86	1.28 ± 0.45 vs 1.23 ± 0.40	6.63 ± 4.01 years vs 6.09 ± 3.85 years	Wu Qin Xi	MBE	Seated Stretching	ST	90 min	3 times/week	24 weeks	NA	Supervision	Home-based	PDSS
68.17 ± 2.27 vs 66.52 ± 2.13	2.2 ± 0.21 vs 2.1 ± 0.23	5.45 ± 3.61 years vs 6.15 ± 2.63 years	Baduanjin Qigong + 30 min walking	MME	30 min walking	AE	40 min	4 times/week	6 months	93.75% vs 91.67%	Unsupervised	Home-based	PDSS-2
64.6 ± 9.7 vs 64.4 ± 9.1	2.5 ± 0.5 vs 2.5 ± 0.4	10.0 ± 4.1 years vs 11.6 ± 6.0 years	RT (leg-press, latissimus dorsi pull-down, ankle plantar flexion, chest-press, and half- squat)	RT	Routine care	CON	50 min	2 times/week	3 months	NA	Supervision	Center for Psychobiology and Exercise Studies	PSQI
65.43 ± 7.27 vs 66.41 ± 7.41	2.51 ± 0.55 vs 2.41 ± 0.68	5.00 ± 5.0 vs 4.00 ± 5.5	Baduanjin Qigong	MBE	Routine care	CON	20–30min	3 times/week	12 weeks	90.91% vs 90.91%	Supervision	Hospital-based	PSQI
63.65 ± 6.02 vs 66.59 ± 8.61	1–2	4.97 ± 3.91 years vs 5.66 ± 3.81 years	ST (Entire body) + AE (Walking back and forth at home) + RT (low to medium intensity)	MME	Routine care	CON	10–50 min,accumulated 150 min/week	3–7 times/week	8 weeks	55.10% vs 100%	Supervision	Home-based	PSQI
66.4 ± 8.1 vs 65.9 ± 5.4	1–3	4.25 ± 2.1 years vs 5.33 ± 3.36 years	Six healing sounds’Qigong	MBE	Sham Qigong	CON	15–20 min	2 times/week	12 weeks	NA	Supervision	Home-based	PDSS-2
66.13 ± 5.66 vs 65.27 ± 4.96	NA	NA	AE (walking on treadmill)	AE	Usual physical therapy	CON	40 min	3 times/week	3 months	NA	NA	NA	Insomnia Severity Index (ISI)
68.53 ± 1.90 vs 67.77 ± 1.72	2 ± 2.2 vs 2 ± 1.2	4.68 ± 0.43 years vs 4.00 ± 0.39 years	Tai Chi (simplified Tai Chi training adapted from Yang style) + Routine exercise	MME	Routine exercise	CON	40–50 min	3 times/week	12 weeks	94.74% vs 86.36%	Supervision	Hospital-based	PDSS
72.07 ± 8.33 vs 69.80 ± 6.90 vs 67.13 ± 8.33	1–4	4.27 ± 3.31 years vs 4.80 ± 3.05 years vs 5.87 ± 2.72 years	Tai Chi (24-Form Simplified Tai Chi Chuan)	MBE	Routine care	CON	(a) 30min (b) 45min	3 times/week	24 weeks	79.17% vs 69.44% vs 100%	Supervision	Hospital-based	PSQI
62.7 ± 5.51 vs 61.5 ± 5.53 vs 62.8 ± 6.14	1–2.5	5.13 ± 3.11 years vs 5.44 ± 3.96 years vs 5.91 ± 4.01 years	(a) Tai Chi (standardized Yi Tai Chi) (b) brisk walking (50%–60% of the maximum heart rate)	(a) MBE (b) AE	Routine care	CON	60 min	2 times/week	12 month	(a) 100% (b) 54.84% vs 53.13%	Supervision	Hospital-based	Epworth Sleepiness Scale (ESS)
59.45 ± 9.37 vs 59.35 ± 8.13 vs 62.13 ± 8.21	1–2.5	39.15 ± 11.65 months vs 39.15 ± 11.65 months vs 36.80 ± 11.23 months	(a) Garba Dance (b) physical therapy (PNF)	(a) AE (b) ST	Routine care	CON	60 min	5 times/week	12 weeks	(a) 85% (b) 85% vs 100%	Supervision	Hospital-based	PDSS-2
67.59 ± 4.94 vs 69.87 ± 3.42	NA	NR	Yijinjing	MBE	Conventional rehabilitation training	CON	30min	5 times/week	8 weeks	92.4% vs 90.7%	Supervision	Hospital-based	PSQI
70.47 ± 7.13 vs 71.77 ± 6.65	1–3	2–14 years vs 3–12 years	Resistance band exercise (Three items for upper limb movement and two items for lower limb movement)	RT	Routine care	CON	20–30min	3 times/week	30 day	NA	Supervision	Hospital-based	PSQI

RCT, Randomized controlled trial; NA, not applicable; AE, aerobic exercise; MME, Multimodal exercise; MBE, mind -body Exercise; RT, resistance training; ST, Stretching training; NR, not reported; vs: versus.

### 2.4 Quality assessment

Risk of bias for the 16 randomized controlled trials was independently assessed by two investigators (Z.D. and W.X.) using the Cochrane RoB 2 tool. In cases of inconsistency, a third reviewer (H.C.) was consulted to reach consensus. The assessment covered domains related to the randomization procedure, adherence to assigned interventions, completeness of outcome data, outcome measurement, and selective reporting. An overall judgment for each study was subsequently assigned as low risk, some concerns, or high risk. Additionally, the certainty of evidence for the primary outcome was evaluated using the GRADE (Grading of Recommendations Assessment, Development and valuation) framework, which classifies evidence as high, moderate, low, or very low.

### 2.5 Statistical analysis

Using Stata (version 19.0), we performed network meta-analysis. A network plot was created to visualize direct comparisons among exercise modalities, where nodes (scaled by sample size) represent interventions and connecting lines (weighted by study count) indicate direct comparisons.

A p-value > 0.05 was considered to indicate no inconsistency between direct and indirect evidence; therefore, a consistency model was applied. Otherwise, an inconsistency model was used. A random-effects model was employed to account for between-study heterogeneity.

Effect sizes were expressed as standardized mean differences (SMD) with 95% confidence intervals for continuous outcomes (pre-post changes in sleep quality scales). Pairwise comparison results were presented in a league table. We obtained intervention rankings via the surface under the cumulative ranking (SUCRA) curve, where SUCRA scores span 0% to 100%; higher scores reflect a greater chance of being among the most effective interventions. Such rankings, however, require cautious interpretation when clinically meaningful differences between interventions are absent. Heterogeneity was assessed using Cochran’s Q test (*p* < 0.10 indicating significant heterogeneity) and the I² statistic (interpreted as: 0–40% low, 40–60% moderate, 60–75% substantial, 75–100% considerable). We explicitly explored consistency versus inconsistency models (*p* < 0.05 indicating inconsistency) using the design-by-treatment interaction model and node-splitting analysis. To evaluate the transitivity assumption, a network meta-regression was performed using mean age as a potential effect modifier.

For publication bias assessment, we generated comparison-adjusted funnel plots and visually checked them for symmetry. Egger’s test was additionally used to quantify asymmetry, with a p−value above 0.05 indicating no significant publication bias.

## 3 Results

### 3.1 Study selection

**Figure 1:**
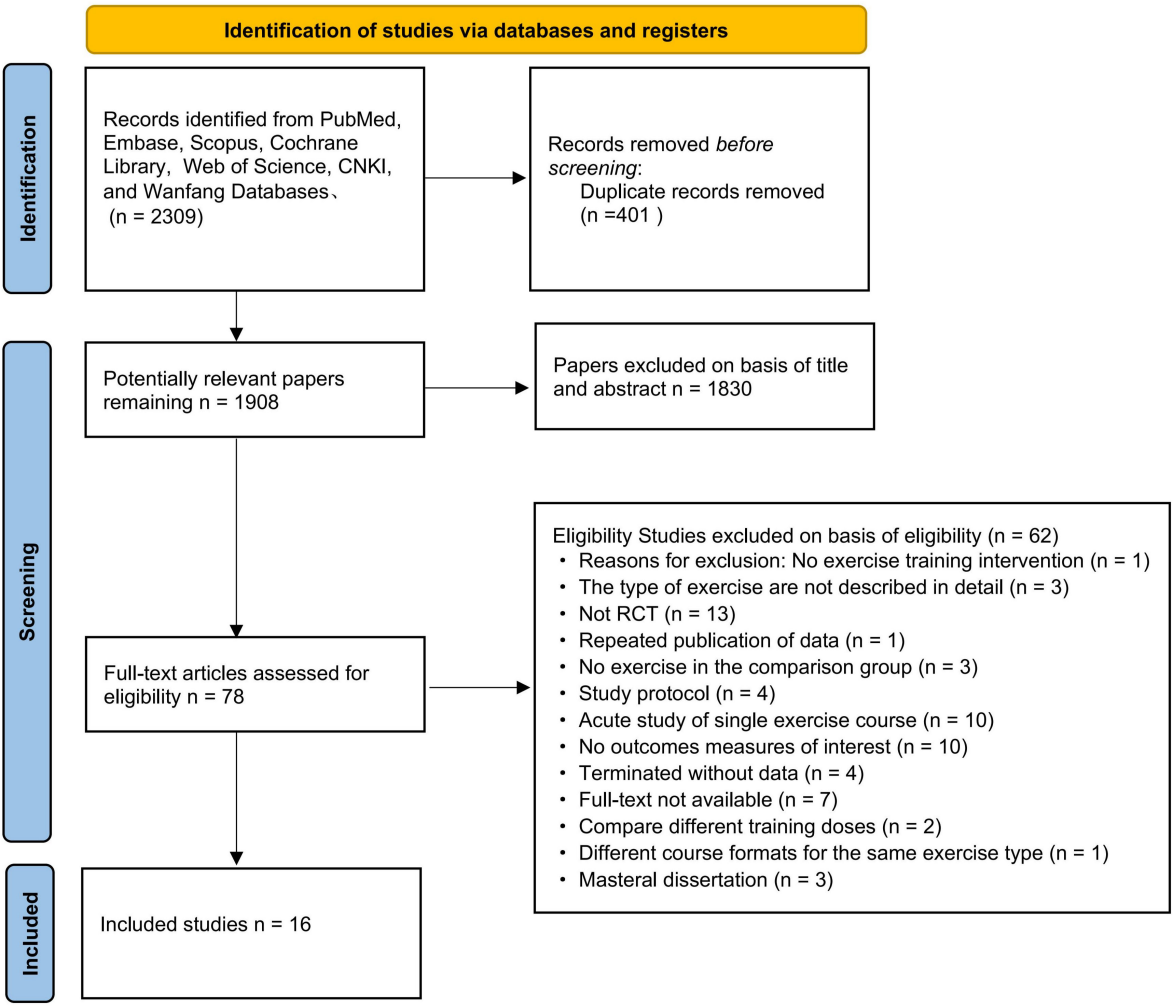
PRISMA flow diagram of the search process for studies.

The investigators (Z.D. and W.X.) each used a comparable method to evaluate the titles and abstracts of the references obtained through the implementation of the aforementioned search technique. Our initial database search yielded 2309 records. Following stepwise screening, 16 trials met the eligibility criteria for the meta-analysis. **Figure 1** presents the PRISMA flowchart, which details the study selection process. The basic characteristics of the 16 included RCTs are summarized in **Table 1**. Inter-rater reliability for study selection was high, with a Cohen’s kappa (κ) of 0.94 (95% CI: 0.89–0.99). A total of 932 participants were enrolled across all trials. The studies were conducted in the United States, China, Brazil, Egypt, and India. All trials were randomized controlled trials, most of which employed a single-blind design, while three used an assessor-blind design. Overall, 58.9% of participants were male and 41.1% were female. The mean age of participants ranged from 59.4 to 72.1 years across studies. Disease duration ranged from 4.0 to 11.6 years, and the Hoehn and Yahr stage was predominantly between 1 and 3. Training frequency ranged from 2 to 5 times per week (mean, 3.1 times/week), and intervention duration ranged from 8 weeks to 12 months. Session duration varied from 15 to 90 min.

### 3.2 Risk of bias assessment

**Figure 2:**
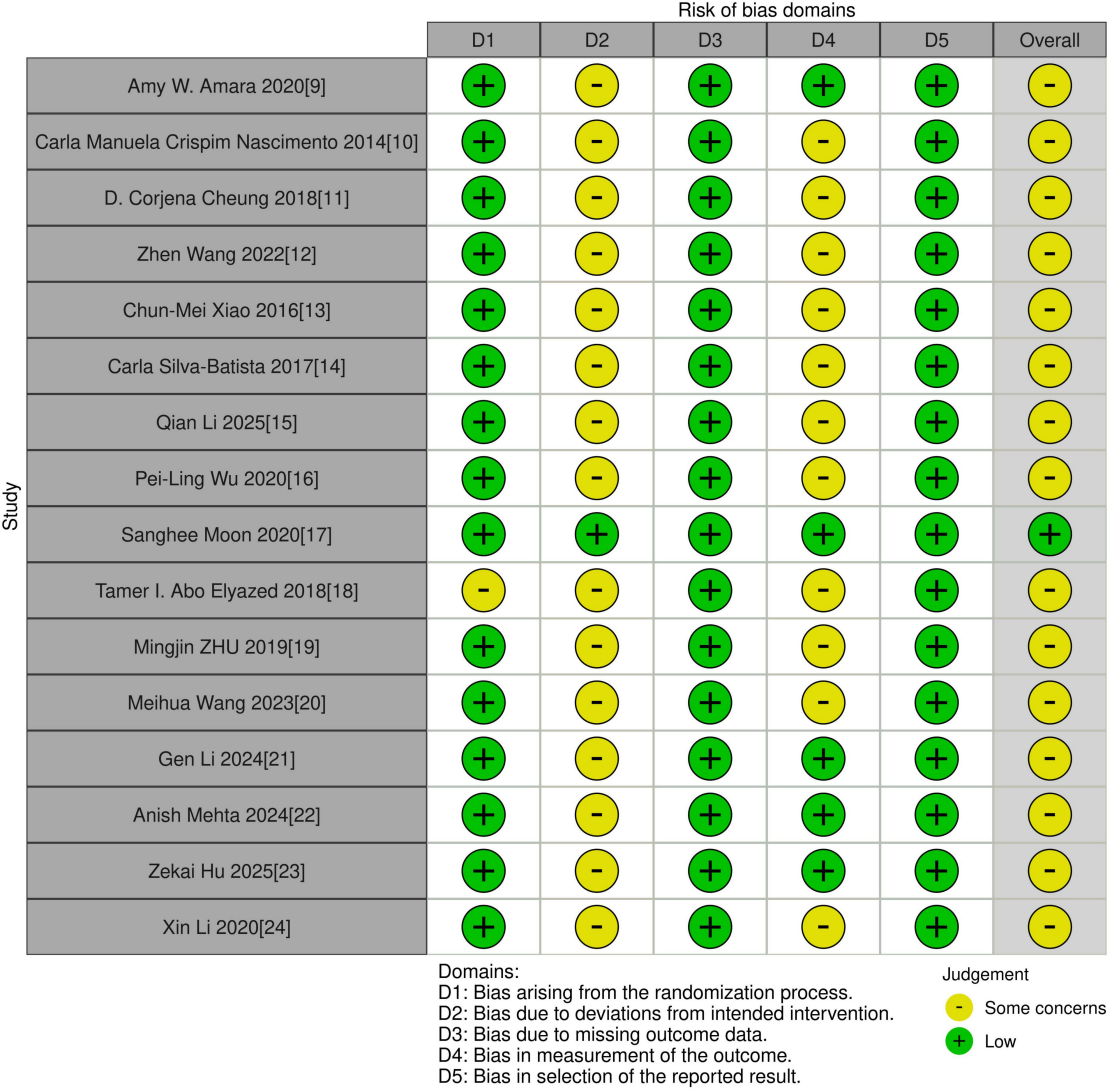
ROB2 risk of bias plot.

Sixteen RCTs were assessed for risk of bias, one study was considered low risk across all five domains, reflecting high methodological quality (Moon et al., 2020). Inter-rater agreement for risk of bias assessment reached a Cohen’s kappa of 1.00. The remaining 15 studies were judged to have some concerns overall, primarily due to the inherent limitation of exercise interventions precluding blinding of participants and personnel. Four studies were rated low risk for measurement bias, as they used assessor-blind designs or objective measurements such as polysomnography (Amara et al., 2020; Li et al., 2024; Mehta et al., 2024; Hu et al., 2025). One study had some concerns regarding randomization due to insufficient description of the randomization method (Abo-Elyazed, 2018). All studies demonstrated adequate management of missing outcome data and selective reporting. Importantly, none of the included RCTs were considered high risk for bias. The detailed risk of bias assessment for each RCT is presented in **Figure 2**.

### 3.3 Network meta-analysis

**Figure 3:**
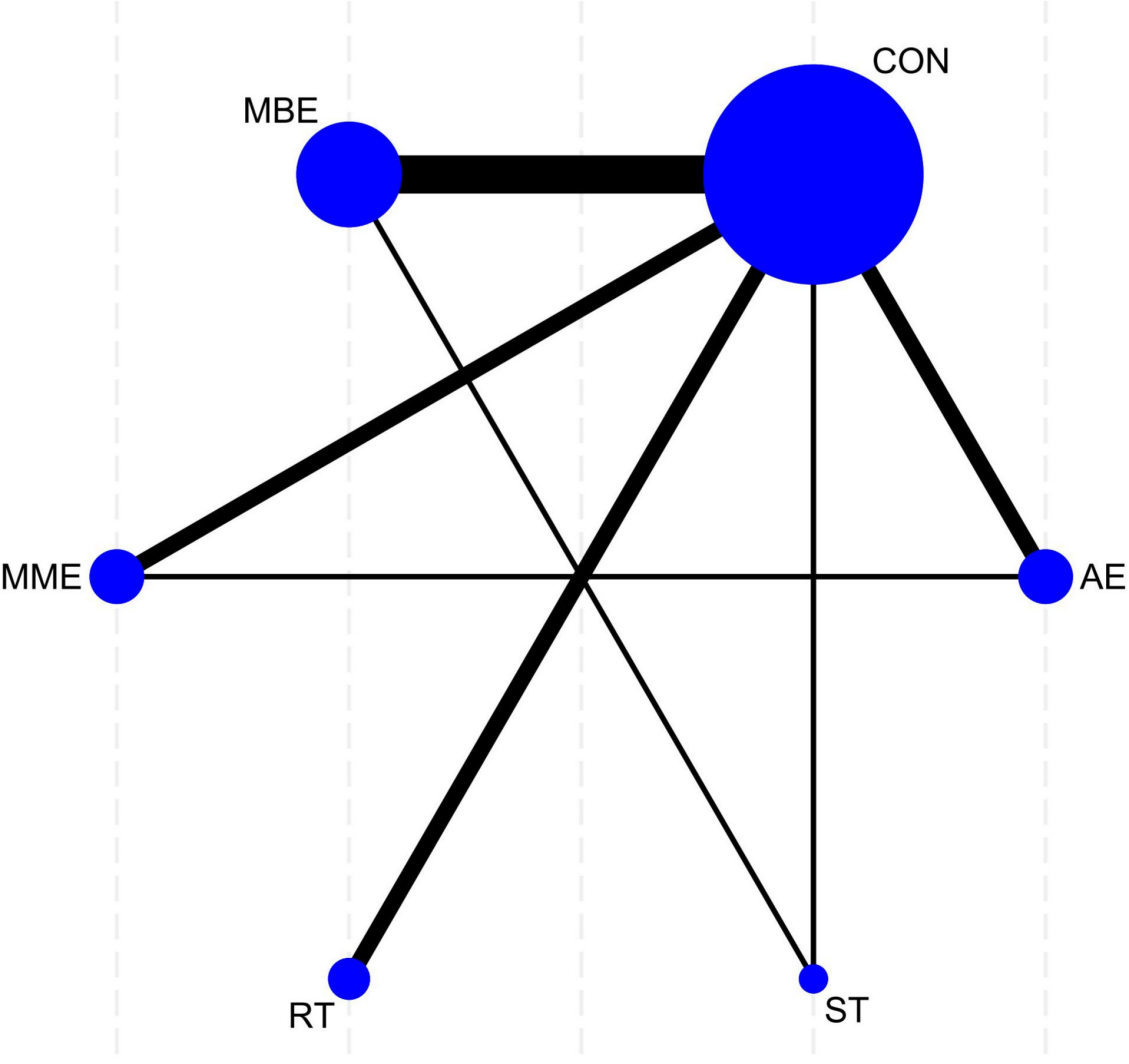
Network diagram.

**Figure 3** displays the network plot summarizing the available evidence on various exercise interventions for sleep quality in people with PD. In the network plot, the size of each node reflects the total number of participants assigned to the corresponding intervention, while the width of the connecting edges indicates the number of direct comparisons between interventions. As shown in the network, The most commonly evaluated interventions included MBE, RT, MME, and aerobic exercise (AE), based on the available direct comparison. Compared with CON, AE yielded an SMD of -0.94 (95% CI: -1.82 to -0.07) for sleep improvement. Detailed pairwise results are in **Table 2**. The global inconsistency test showed no significant difference between the consistency and inconsistency models (χ² = 4.70, *p* = 0.0956). Meta-regression analysis indicated that mean age did not significantly influence the treatment effect (*p* = 0.67), supporting the transitivity assumption (**Table 3**). However, node-splitting analysis revealed local inconsistencies in three comparisons: CON vs AE, CON vs MME, and AE vs MME (*p* < 0.05) (**Table 4**).

**Table 2:** League table.

	AE	MME	RT	ST	MBE	CON
AE	—					
MME	-0.15 (-1.19, 0.88)	—				
RT	-0.32 (-1.62, 0.98)	-0.17 (-1.45, 1.12)	—			
ST	-0.42 (-1.91, 1.07)	-0.26 (-1.73, 1.21)	-0.10 (-1.65, 1.46)	—		
MBE	-0.44 (-1.50, 0.63)	-0.29 (-1.32, 0.75)	-0.12 (-1.27, 1.03)	-0.02 (-1.23, 1.18)	—	
CON	-0.94 (-1.82, -0.07)*	-0.79 (-1.63, 0.05)	-0.62 (-1.60, 0.36)	-0.53 (-1.73, 0.68)	-0.51 (-1.11, 0.10)	—

AE, Aerobic exercise; MME, multimodal exercise; RT, resistance training; ST, stretching training; MBE, mind-body exercise.

*Data are mean difference (95% CI). For CON vs. AE: -0.94 (-1.82, -0.07). Negative values favor AE; CI not crossing zero implies p < 0.05.

**Table 3:** Network meta-regression results with mean age as a covariate.

Covariate	Coefficient	95% CI	P-value	τ²	*I*²
Mean age (per 1 year)	-0.021	(-0.118, 0.076)	**0.67**	0.23	56.2%
Constant	-0.94	(-1.58, -0.30)	0.004	–	–

SUCRA rankings after adjusting for age.

AE: 90.2%, MME: 72.0%, RT: 62.5%, MBE: 41.8%, ST: 37.6%, CON: 5.9%.

Meta-regression analysis indicated that mean age did not significantly influence the treatment effect (p = 0.67), supporting the transitivity assumption.

**Table 4:** Node-splitting analysis for each treatment comparison.

Side	Direct coef.	Std. err.	Indirect coef.	Std. err.	Difference coef.	Std. err.	P>z	tau
A B	1.36521	.4523134	-.6219843	.8164934	1.987194	.9331224	0.033*	.667239
A D	-1.012347	.7045362	.9800926	.616995	-1.99244	.9365117	0.033*	.6673912
B C	-.4561601	.330916	-1.17915	1.225929	.7229904	1.269808	0.569	.80281
B D	-.3815783	.4206685	-2.375126	.8369071	1.993548	.9366985	0.033*	.6674352
B E	-.6240301	.4998718	-1.884963	36.53341	1.260933	36.53663	0.972	.775066
B F	-.8711748	.8796911	-.151113	.9186096	-.7200618	1.271891	0.571	.8029
C F	.305973	.8571844	-.4150049	.939744	.7209779	1.27196	0.571	.8029131

A, AE, B, CON, C, MBE, D, MME, E, RT, F, ST.

Node‑splitting coefficients are presented as the effect of treatment versus control (positive values favor control, *p<0.05.

### 3.4 SUCRA probability ranking

**Figure 4:**
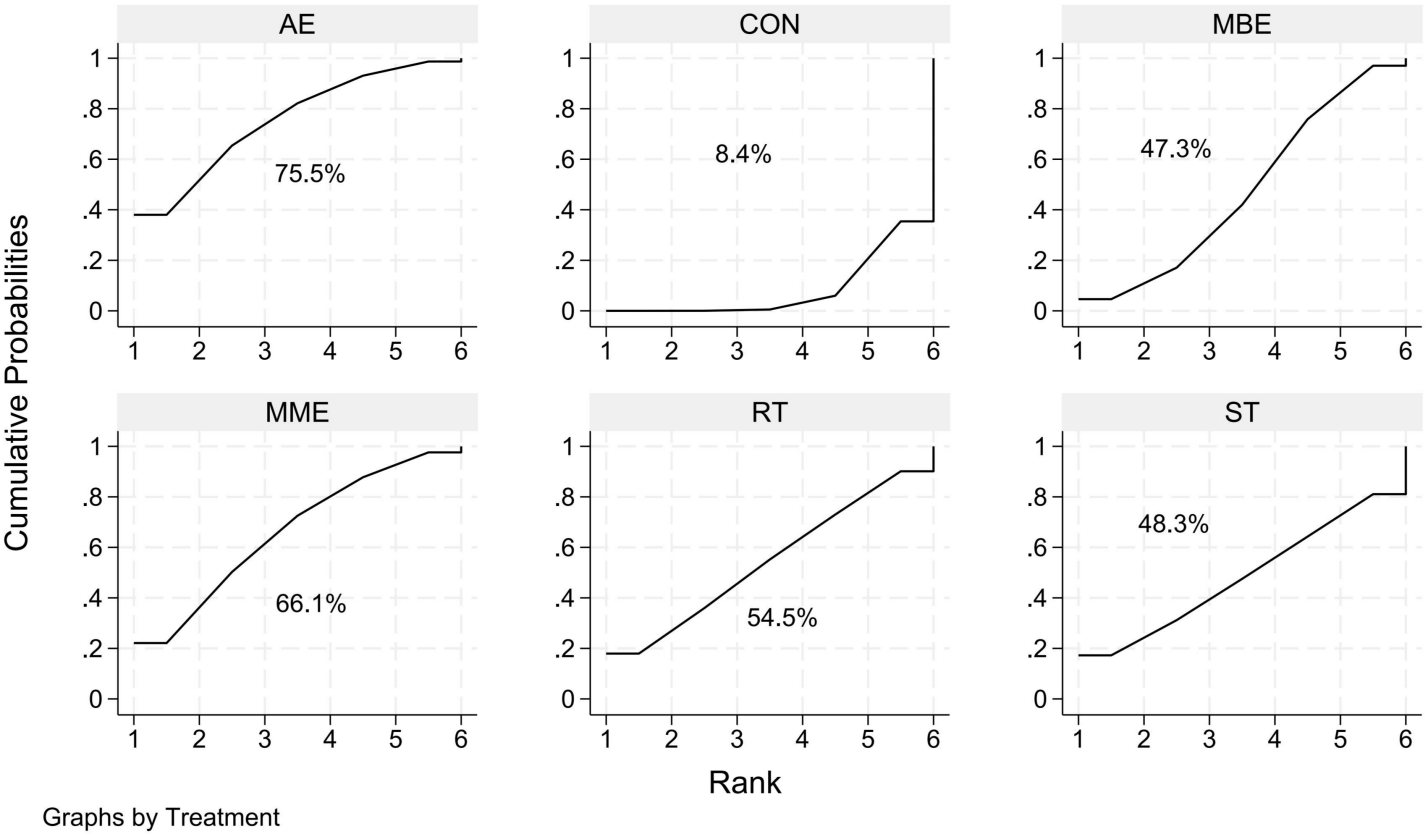
Surface under the cumulative ranking curve.

**Figure 4** presents the SUCRA plots along with their corresponding numerical values for the various exercise interventions. The SUCRA scores provide a probabilistic ranking of each intervention’s relative effectiveness in improving sleep quality. Among the six interventions evaluated, AE achieved the highest SUCRA score of 75.5%, indicating the greatest probability of being the most effective modality. MME ranked second with a SUCRA score of 66.1%, followed by RT at 54.5%. ST and MBE showed moderate rankings, with SUCRA scores of 48.3% and 47.3%, respectively. The CON had the lowest SUCRA score of 8.4%, confirming that all active exercise interventions were superior to no exercise. Overall, the relative effectiveness of different exercise methods in improving sleep quality, based on SUCRA rankings, is as follows: AE (75.5%) > MME (66.1%) > RT (54.5%) > ST (48.3%) > MBE (47.3%) > CON (8.4%).

### 3.5 Publication bias

**Figure 5:**
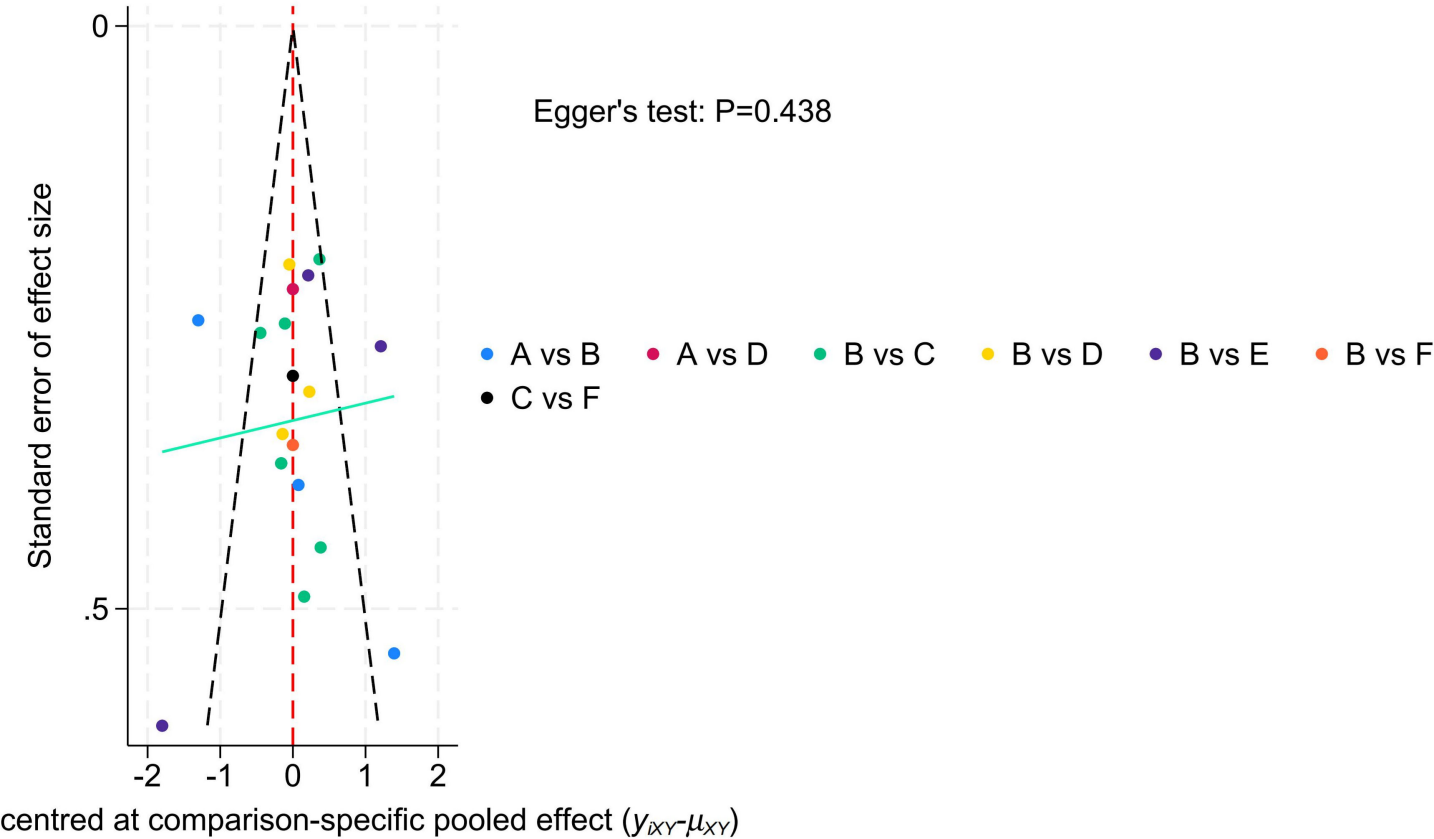
Funnel plot.

Funnel plots were constructed to visually assess the differences in mean changes for all outcome measures between the active interventions and the control group. Across all plots, the majority of data points were symmetrically distributed on either side of the central vertical line. Egger’s test was additionally performed to quantitatively evaluate potential publication bias, which yielded no evidence of significant asymmetry (P = 0.438). These results suggest no evidence of substantial publication bias among the comparisons included in this network meta-analysis (**Figure 5**).

### 3.6 Quality of evidence (GRADE)

The overall certainty of evidence for the effect of AE on sleep quality was rated as ‘Very low’ (**Table 5**). Downgrading was primarily due to the lack of blinding (risk of bias) and serious inconsistency in some loops.

**Table 5:** GRADE evidence.

Comparison	Number of studies	Within study bias	Reporting bias	Indirectness	Imprecision	Heterogeneity	Incoherence	Certainty of evidence
AE: CON	3	Major concerns	No concerns	No concerns	No concerns	Major concerns	No concerns	Very low
AE: MBE	0	No concerns	No concerns	Major concerns	Major concerns	Major concerns	No concerns	Very low
CON: MBE	7	Major concerns	No concerns	No concerns	Major concerns	Major concerns	No concerns	Very low
AE: MME	1	Major concerns	No concerns	Some concerns	Major concerns	Major concerns	No concerns	Very low
CON: MME	3	Major concerns	No concerns	No concerns	No concerns	Major concerns	No concerns	Very low
MBE: MME	0	No concerns	No concerns	Major concerns	Major concerns	Major concerns	No concerns	Very low

AE, Aerobic exercise; MME, multimodal exercise; MBE, mind-body exercise.

## 4 Discussion

This network meta-analysis integrated evidence from randomized controlled trials to evaluate the comparative efficacy of six exercise modalities in improving sleep quality among people with PD. AE ranked highest based on SUCRA probabilities, followed by MME, RT, ST, MBE, and control conditions. The league table of pairwise comparisons revealed that only AE achieved a statistically significant improvement relative to the control group (SMD −0.94, 95% CI −1.82 to −0.07). Therefore, AE is recommended as the optimal exercise modality to enhance sleep quality in people with PD.

These findings are consistent with the wider body of literature on the beneficial effects of exercise in PD and in older adult populations. Aerobic modalities consistently emerge as highly effective for subjective sleep quality metrics, where AE often achieves top probabilistic rankings (Gao et al., 2024). In PD cohorts, AE has been linked to enhanced motor function, better cognitive performance, and a reduced overall non-motor symptom burden (Xu G. et al., 2025). Network meta-analyses of physical exercise in PD further demonstrate beneficial effects of multiple exercise modalities on motor signs and quality of life, with aerobic approaches frequently ranking highest (Ernst et al., 2024).

AE may indirectly optimize sleep by supporting emotional regulation (Ge et al., 2025). Specifically, AE has been suggested to enhance prefrontal cortex (PFC)—particularly its dorsolateral and orbitofrontal areas—while improving amygdala-PFC functional connectivity (Johansson et al., 2022). Following chronic AE, positive amygdala-orbitofrontal connectivity is strengthened. This top-down regulatory mechanism effectively suppresses excessive amygdala reactivity to negative emotions, reducing the intensity of anxiety, depression, and tension, thereby decreasing emotion-related arousal and rumination that interfere with sleep.

AE may contribute to structural plasticity of the uncinate fasciculus, a key limbic white matter tract connecting the prefrontal cortex with temporal and amygdala regions and serving as a central pathway for emotional integration. Exercise-induced neuroplasticity improves microstructural integrity of the uncinate fasciculus, enhancing prefrontal top-down control over limbic emotional signals (Chen et al., 2022). In people with PD, baseline reductions in amygdala-prefrontal connectivity and uncinate fasciculus integrity are often accompanied by mood disturbances (Carey et al., 2023; Rashidi et al., 2023), which in turn mediate difficulties in sleep onset, increased nocturnal awakenings, and diminished subjective sleep quality. Through this neural network remodeling, AE not only directly alleviates comorbid mood symptoms but also reduces the disruption of sleep continuity by emotional hyperarousal, leading to significant improvements in global PSQI scores. This emotional regulation mechanism constitutes a key mediating pathway underlying AE’s leading position in sleep quality rankings, with potentially greater effects in PD subgroups characterized by earlier Hoehn-Yahr stages and higher comorbid emotional burden.

Additionally, PD is commonly characterized by neuroendocrine dysregulation, including elevated cortisol levels and reduced melatonin secretion, both of which contribute to sleep-wake cycle disturbances (Luthra et al., 2022; Hu et al., 2023). Chronic AE has been shown to normalize these parameters (De Nys et al., 2022). A meta-analysis examining different exercise interventions found that AE reduced cortisol levels to the greatest extent compared with resistance training in patients with major depressive disorder (Luthra et al., 2025), suggesting that AE possesses unique advantages in regulating the hypothalamic-pituitary-adrenal axis. Concurrently, AE enhances melatonin secretion, thereby further improving sleep quality through circadian rhythm regulation (Shen et al., 2025).

The aerobic protocols in the included studies typically involved 40–60-minute sessions performed at moderate intensity, 3–5 times per week, for at least 3 months (most commonly 12–24 weeks), using accessible modalities such as treadmill walking or dance. These parameters align well with current recommendations for achieving sustainable benefits in PD.

The symmetry of the funnel plot and the non-significant Egger’s test (P = 0.438) suggest minimal publication bias, enhancing confidence in the pooled estimates. However, limitations include potential heterogeneity in sleep assessment tools, variable intervention fidelity, small sample sizes in some pairwise comparisons, and under-representation of objective sleep measures. Direct head-to-head trials remain scarce, and long-term adherence data are limited. Furthermore, the lack of statistical significance for non-aerobic modalities should be interpreted with caution, as it may stem from insufficient statistical power rather than a true absence of effect. Additionally, We acknowledge that our analysis primarily reflects evidence from patients with mild-to-moderate PD (Hoehn & Yahr stages 1–3), as very few trials included advanced-stage patients (e.g., stage 4). Patients with severe motor impairment often face mobility and safety limitations that may restrict their participation in aerobic exercise. Therefore, the generalizability of our recommendations to advanced-stage PD is uncertain and requires further investigation. Finally, a limitation is the inability to apply Minimal Clinically Important Difference (MCID) thresholds. This is due to the lack of validated MCID values for sleep outcomes in the PD population, the use of inconsistent measurement tools across studies, and insufficient reported data to calculate individual-level clinical significance.

## 5 Conclusion

In conclusion, this NMA positions aerobic exercise as the most promising modality for improving sleep quality in patients with mild-to-moderate PD. Multimodal exercise ranks second but requires further validation. Clinicians should prioritize individualized aerobic exercise prescriptions as a safe, non-pharmacological adjunct to improve sleep quality. Future large-scale RCTs with objective sleep outcomes and subgroup analyses are warranted. We note that evidence for advanced-stage PD is limited, and generalizability to this population requires further investigation.

## Data Availability

The original contributions presented in the study are included in the article/**Supplementary Material**. Further inquiries can be directed to the corresponding author.
