# Supplementary material for: Efficacy of different exercise modalities for sleep quality in Parkinson’s disease: a systematic review and network meta-analysis
Source: Front Physiol. 2026 Jun 11;17:1854427. doi: 10.3389/fphys.2026.1854427 (PMC13293900; doi:10.3389/fphys.2026.1854427)
Supplement: Supplementary file 1 [file DataSheet1.pdf]

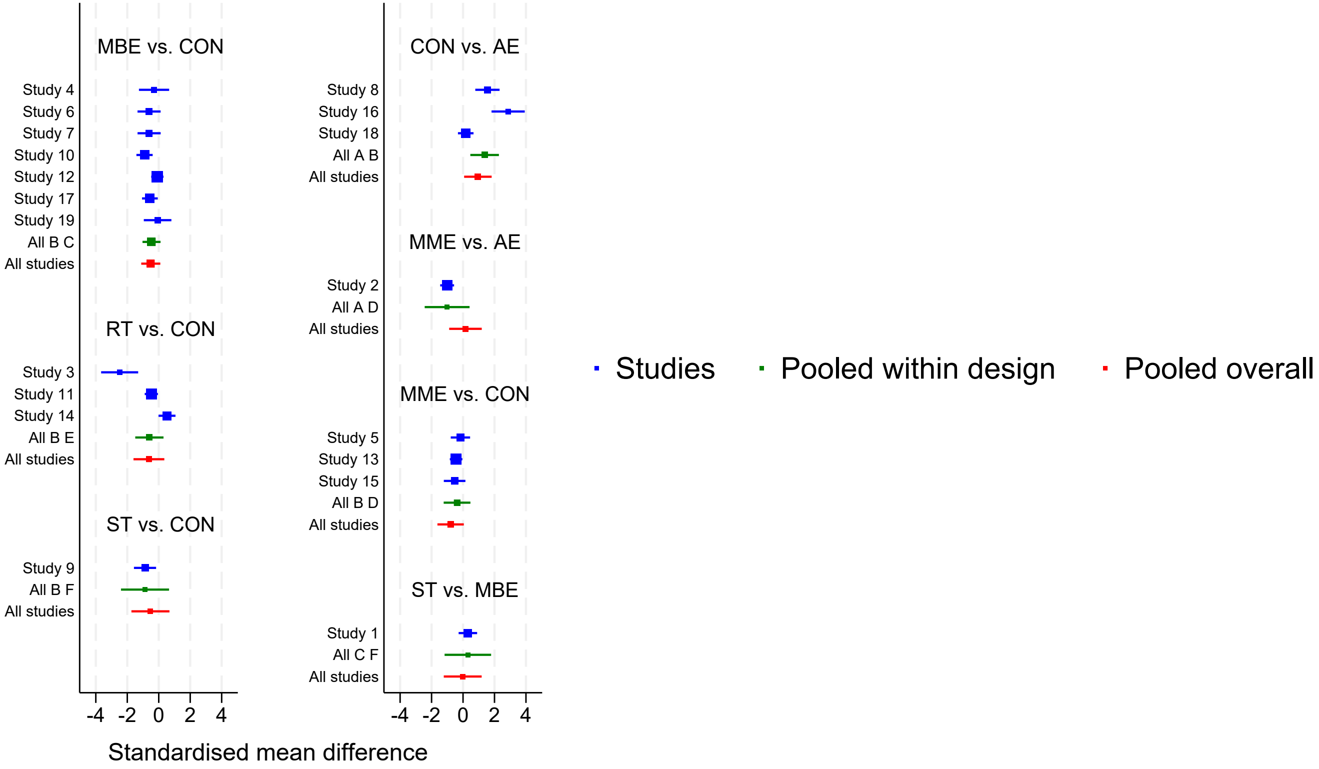

Test of consistency: chi2(2)=4.70, P=0.096
