# Supplementary material for: Efficacy of different exercise modalities for sleep quality in Parkinson’s disease: a systematic review and network meta-analysis
Source: Front Physiol. 2026 Jun 11;17:1854427. doi: 10.3389/fphys.2026.1854427 (PMC13293900; doi:10.3389/fphys.2026.1854427)
Supplement: Supplementary file 2 [file Table1.docx]

| (((((((parkinson's disease[MeSH Terms]) OR (parkinson[MeSH Terms])) OR (parkinson disease[MeSH Terms])) OR (parkinson's[MeSH Terms])) OR (parkingson[MeSH Terms])) OR (parkinson's desease[MeSH Terms])) OR (parkson disease[MeSH Terms])) OR (parkinson disaese[MeSH Terms]) |
| --- |
| (((((((Sleep[MeSH Terms]) OR (sleep-disordered[Title/Abstract])) OR (sleep[Title/Abstract])) OR ("sleep quality"[Title/Abstract])) OR ("sleep status"[Title/Abstract])) OR ("sleep stages"[Title/Abstract])) OR (slumber[Title/Abstract])) OR (nap[Title/Abstract]) |
| ((( "Exercise"[Mesh] OR "Exercise Therapy"[Mesh] OR "Resistance Training"[Mesh] OR "Yoga"[Mesh] OR "Tai Ji"[Mesh] OR "Dance Therapy"[Mesh] OR "Muscle Stretching Exercises"[Mesh] OR "Weight-Bearing"[Mesh] OR "Physical Therapy Modalities"[Mesh] OR "exercise*"[tiab] OR "train*"[tiab] OR "physical activit*"[tiab] OR "strength training"[tiab] OR "balance training"[tiab] OR "neuromuscular training"[tiab] OR "proprioceptive training"[tiab] OR "postural training"[tiab] OR "dual-task training"[tiab] OR "aquatic exercis*"[tiab] OR "water-based exercis*"[tiab] OR "yoga"[tiab] OR "tai chi"[tiab] OR "taiji"[tiab] OR "qigong"[tiab] OR "pilates"[tiab] OR "dance"[tiab] OR "vibration training"[tiab] OR "sensorimotor training"[tiab] )) OR ("aerobic exercise*"[tiab])) OR ("endurance training"[tiab]) |

Search strategies
