# Supplementary material for: Efficacy of different exercise modalities for sleep quality in Parkinson’s disease: a systematic review and network meta-analysis
Source: Front Physiol. 2026 Jun 11;17:1854427. doi: 10.3389/fphys.2026.1854427 (PMC13293900; doi:10.3389/fphys.2026.1854427)
Supplement: Supplementary file 3 [file Table2.docx]

## Study-level effect sizes and covariates（based on change scores, r = 0.5）

| Study | Intervention | Comparator | Outcome | *g* (Hedges' g) | SE(g) | Mean_age_overall |
| --- | --- | --- | --- | --- | --- | --- |
| Zhen Wang 2022 | MBE | ST | PDSS | -0.306 | 0.300 | 68.39 |
| Chun-Mei Xiao 2016 | MME | AE | PDSS-2 | -1.223 | 0.225 | 67.35 |
| Carla Silva-Batista 2017 | RT | CON | PSQI | -2.891 | 0.673 | 64.50 |
| Sanghee Moon 2020 | MBE | CON | PDSS-2 | -0.711 | 0.498 | 66.15 |
| Tamer I. Abo Elyazed 2018 | AE | CON | ISI | -2.962 | 0.529 | 65.70 |
| Mingjin ZHU 2019 | MME | CON | PDSS | -0.286 | 0.317 | 68.15 |
| Meihua Wang 2023 (a) | MBE | CON | PSQI | -0.802 | 0.379 | 70.94 |
| Meihua Wang 2023 (b) | MBE | CON | PSQI | -0.775 | 0.378 | 68.47 |
| Gen Li 2024 (a) | MBE | CON | ESS | -0.425 | 0.251 | 62.10 |
| Gen Li 2024 (b) | AE | CON | ESS | -0.102 | 0.254 | 62.15 |
| Anish Mehta 2024 (a) | AE | CON | PDSS-2 | -1.387 | 0.423 | 60.79 |
| Anish Mehta 2024 (b) | ST | CON | PDSS-2 | -0.754 | 0.396 | 60.74 |
| Zekai Hu 2025 | MBE | CON | PSQI | -0.815 | 0.262 | 68.73 |
| Xin Li 2020 | RT | CON | PSQI | -0.742 | 0.215 | 71.12 |
| Qian Li 2025 | MBE | CON | PSQI | -0.441 | 0.202 | 65.92 |
| Pei-Ling Wu 2021 | MME | CON | PSQI | -0.498 | 0.203 | 65.12 |
| Amy W. Amara 2020 | RT | CON | PSQI | -0.526 | 0.274 | 65.58 |
| Carla Nascimento 2014 | MME | CON | MSQ | -0.408 | 0.347 | 67.05 |
| Corjena Cheung 2018 | MBE | CON | PDSS | -0.312 | 0.454 | 64.65 |

Abbreviations: A negative g indicates that the change in the intervention group was superior to that in the control group (i.e., the intervention was effective). All calculations were based on change scores, assuming a pre‑post correlation coefficient of r=0.5.
