# Supplementary material for: Efficacy of different exercise modalities for sleep quality in Parkinson’s disease: a systematic review and network meta-analysis
Source: Front Physiol. 2026 Jun 11;17:1854427. doi: 10.3389/fphys.2026.1854427 (PMC13293900; doi:10.3389/fphys.2026.1854427)
Supplement: Supplementary file 4 [file Table3.docx]

## **Distribution of potential effect modifiers across different intervention comparisons**

| Intervention comparison | Studies (k) | Mean age (years) | Hoehn‑Yahr stage | Disease duration (years) |
| --- | --- | --- | --- | --- |
| AE vs. CON | 3 | 62.7 – 66.1 | 1 – 2.5 | 5.0 – 6.1 |
| RT vs. CON | 3 | 64.6 – 70.5 | 1 – 3 | 4.0 – 11.6 |
| MBE vs. CON | 6 | 59.4 – 72.1 | 1 – 4 | 4.3 – 5.9 |
| MME vs. CON | 4 | 63.7 – 68.5 | 1 – 2.2 | 4.0 – 5.7 |
| ST vs. CON | 1 | 59.4 / 62.1 | 1 – 2.5 | 36.8 / 39.2 |

Abbreviations: The ranges of all covariates overlapped across different comparison groups, with no systematic differences.
